# Absence of Mal/TIRAP Results in Abrogated Imidazoquinolinones-Dependent Activation of IRF7 and Suppressed IFNβ and IFN-I Activated Gene Production

**DOI:** 10.3390/ijms21238925

**Published:** 2020-11-25

**Authors:** Ewa Leszczyńska, Edyta Makuch, Małgorzata Mitkiewicz, Izabella Jasyk, Miwako Narita, Sabina Górska, Tomasz Lipiński, Jakub Siednienko

**Affiliations:** 1 Bioengineering Research Group, Łukasiewicz Research Network–PORT Polish Center for Technology Development, 54-066 Wroclaw, Poland; ewka.kowalczyk@gmail.com (E.L.); Izabella.Jasyk@port.lukasiewicz.gov.pl (I.J.); Tomasz.Lipinski@port.lukasiewicz.gov.pl (T.L.); 2 Laboratory of Microbiome Immunobiology, Ludwik Hirszfeld Institute of Immunology and Experimental Therapy, Polish Academy of Sciences, 53-114 Wroclaw, Poland; Edyta.Makuch@port.lukasiewicz.gov.pl (E.M.); malgorzata.mitkiewicz@hirszfeld.pl (M.M.); sabina.gorska@hirszfeld.pl (S.G.); 3 Laboratory of Hematology and Oncology, Niigata University, Niigata 950-2181, Japan; naritami@clg.niigata-u.ac.jp

**Keywords:** R848, R837, Toll-like receptor-7, Mal, IRF7, Interferon-beta, IP-10

## Abstract

Activation of TLR7 by small imidazoquinoline molecules such as R848 or R837 initiates signaling cascades leading to the activation of transcription factors, such as AP-1, NF-κB, and interferon regulatory factors (IRFs) and afterward to the induction of cytokines and anti-viral Type I IFNs. In general, TLRs mediate these effects by utilizing different intracellular signaling molecules, one of them is Mal. Mal is a protein closely related to the antibacterial response, and its role in the TLR7 pathways remains poorly understood. In this study, we show that Mal determines the expression and secretion of IFNβ following activation of TLR7, a receptor that recognizes ssRNA and imidazoquinolines. Moreover, we observed that R848 induces Mal-dependent IFNβ production via ERK1/2 activation as well as the transcription factor IRF7 activation. Although activation of TLR7 leads to NF-κB-dependent expression of IRF7, this process is independent of Mal. We also demonstrate that secretion of IFNβ regulated by TLR7 and Mal in macrophages and dendritic cells leads to the IP-10 chemokine expression. In conclusion, our data demonstrate that Mal is a critical regulator of the imidazoquinolinones-dependent IFNβ production via ERK1/2/IRF7 signaling cascade which brings us closer to understanding the molecular mechanism’s regulation of innate immune response.

## 1. Introduction

The imidazoquinolines are synthetic agonists for Toll-like receptors (TLRs) and include resiquimod, imiquimod, and gardiquimod with potent anti-viral activity. These molecules exert their action in innate immune systems by binding TLR7 and/or TLR8 [1]. The role of imidazoquinolines to stimulate innate immunity indicates its potential to treat viral infections, such as SARS-CoV-2 in early stages of the disease, where activation of innate immunity by a TLR7/8 agonist is of vital importance [2,3].

In general, TLRs are a family of pattern recognition receptors that functional homologs were initially described in *Drosophila melanogaster* [3]. These transmembrane glycoproteins play a crucial role in innate immune response, recognizing various molecular patterns associated with pathogens. TLRs triggered signaling cascades facilitate eradication of invading viruses and bacteria. All TLRs share similar domain structures: extracellular ligand-binding domain, transmembrane region, and an intracellular TIR (Toll-like/Interleukin 1 Receptor) domain responsible for recruiting downstream signaling proteins. So far, 11 TLRs in humans and 13 TLRs in mice have been characterized [4]. According to cellular localization, TLRs fall into two groups: receptors located in the plasma membrane and those located in the endosomal compartment. While the first group members interact with several types of macromolecules such as proteins, saccharides, or lipids, the latter recognizes nucleic acids of bacterial and viral origin. TLR7 has been shown to bind single-stranded RNA (ssRNA) from viruses such as the Influenza virus, HIV-1, or the Epstein–Barr virus [5] and small imidazoquinoline molecules such as R848 or R837 [1]. Recognition of host endogenous molecules by endosomal TLRs is inclined to contribute to autoimmune disease development [6]. Recently, TLR7 has been found to be important also in the immune response to COVID-19. Male patients with severe COVID-19, rare putative loss-of-function variants of X-chromosomal TLR7 were identified that were associated with impaired type I and II IFN responses [7].

Ligand-receptor interaction initiates complex signaling pathways starting with the recruitment of TIR-containing adaptor molecules to the receptor’s intracellular domain where TIR-TIR interaction occurs [4]. Up to date, five different TIR containing proteins have been identified: Myeloid differentiation factor 88 (MyD88), MyD88 adaptor-like (Mal)/TIR adaptor protein (TIRAP), TIR domain-containing adaptor inducing interferon-beta (TRIF) and TRIF-related adaptor molecule (TRAM), and negative regulator: Sterile-alpha and armadillo motif-containing protein (SARM) [4,8]. Similar signaling cascades are activated after TLR engagement and result in the production of interferons (IFNs) and pro-inflammatory cytokines such as IL-6 and chemokines (i.e., IP-10). Apart from TLR3 and TRAM-inducing signaling from TLR4, almost all characterized TLRs employ MyD88 as their primary adaptor protein. Due to subtle structural differences in the receptor’s TIR domain, some of these TLRs require an additional adaptor protein to form a platform for MyD88 binding. Mal has been found essential the signal transduction in several TLRs such as TLR2 and TLR4 [8]. On the other hand, it has been shown that Mal can negatively regulate TLR3 signaling by impeding Poly(I:C)-induced IRF7 activation [9]. It has also been reported the potential involvement of Mal in TLR7 and TLR9 signaling pathways [10,11].

TLR7 activated by imidazoquinolines, has been shown to engage the MyD88-dependent signaling pathway [12]. After MyD88 recruitment, a multiprotein complex is formed. It consists of kinases of the IRAK family: IRAK4, 2, and 1, where IRAK4 interacts with MyD88 through its own death domain (DD). Subsequently, an E3 ubiquitin ligase TRAF6 is recruited, which in turn activates both the NF-κB and MAPK/AP-1 transcription factors leading to proinflammatory cytokine and chemokine expression. The IRF family of transcription factors is also activated after ssRNA recognition by TLR7 and mediates viral-dependent IFN-I activation. After MyD88 recruitment, a complex of IRAK4/IRAK1-TRAF6/TRAF3 and IRF7 is formed leading to IRF7 phosphorylation [13]. Thereafter, the complex dissociates and IRF7 is translocated into the nucleus where it induces transcription of type I interferon (IFNα/β) genes. On the other hand, recent literature reports suggest that not only MyD88 is involved in TLR7 signaling pathways, but also the Mal protein. It has been shown that the response to viruses that activate TLR7 is diminished in Mal-deficient cells [10] and next it has been confirmed Mal is important for endosomal TLR7 signaling [11]. However, the molecular mechanism of the Mal-dependent TLR7 signaling pathways remains unknown.

Here we show for the first time that the adaptor protein Mal is required for R848-induced IFNβ expression via ERK1/2 kinases activation and leads to IP-10 induction. Moreover, we show that following TLR7 activation IRF7 is regulated on two levels: IRF7 gene expression induced by the NF-κB dependent path, and Mal-dependent IRF7 recruitment to IFNβ promoter.

## 2. Results

### 2.1. In Macrophages Mal Positively Regulates IFNβ Induction via ERK1/2 after R848 Treatment

**Figure 1:**
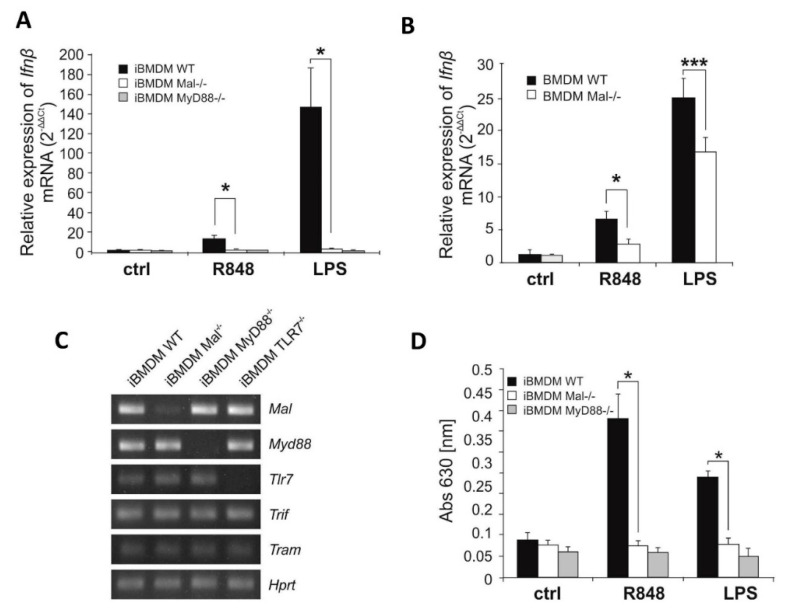
Resiquimod-dependent IFNβ induction is downregulated in Mal-deficient cell. (**A**,**B**) Wild type (WT), Mal-deficient (Mal^−/−^) and MyD88-deficient (MyD88^−/−^) iBMDMs (**A**) and macrophages isolated from bone marrow of wild-type mice (BMDM WT) and Mal-deficient mice (BMDM Mal^−/−^) (**B**) were treated with R848 (100 nM) or LPS (100 ng/mL) for 4 h. Thereafter, total RNA was isolated, converted to first-strand cDNA and used as a template for quantitative real-time RT-PCR as described under “Materials and Methods”. Quantitative real-time PCR was used to assay the expression levels of *Ifnβ*. Experiments were repeated at least three times and data are presented in relative expression units, where *Hprt* was used to normalize all samples. Non-treated cells were assigned an arbitrary value of 1. (**C**) Total RNA was isolated from WT, Mal^−/−^, MyD88^−/−^ and TLR7^−/−^ iBMDMs and converted to first-strand cDNA. This was used as a template for conventional PCR amplifying genes as indicated. Products were resolved as described under “Materials and Methods”. (**D**) WT, Mal^−/−^, and MyD88^−/−^ iBMDMs were treated with R848 (100 nM) or LPS (100 ng/mL) for 16 h. Thereafter, type I IFN was measured by bioassay as described under “Materials and Methods”. Results are representative of at least three independent experiments performed in triplicate (Mean ± S.E.). * *p* ≤ 0.001; *** *p* ≤ 0.05.

Previous studies conducted by our group have shown the ability of Mal protein to down-regulate TLR3 dependent signaling [9]. Specifically, we have shown that Mal suppresses TLR3-mediated IFNβ production via negative regulation of IRF7. To elucidate the role of Mal in TLR7 signaling, we sought to investigate the ability of this adapter to modulate TLR7-mediated cytokine production. To this end, we measured TLR7-mediated *Ifnβ* induction by quantitative PCR. Following quantitative real-time PCR measurements, we demonstrate that stimulation of wild-type (WT) iBMDMs with the TLR7 ligand, R848 resulted in *Ifnβ* gene induction, while significantly lower induction of *Ifnβ* was evident in Mal^−/−^ iBMDMs. Similarly, impaired *Ifnβ* gene induction was evident in Mal^−/−^ iBMDMs when compared with WT controls following LPS stimulation (Figure 1A). As expected, *Ifnβ* gene induction was not evident in MyD88^−/−^ iBMDMs following stimulation with either R848 or LPS (Figure 1A). A similar effect of Mal on *Ifnβ* gene expression was observed in macrophages in vitro differentiated from WT and Mal^−/−^ mice bone marrow (WT and Mal^−/−^ BMDMs) (Figure 1B).

Additionally, Tlr7 knockout iBMDMs were checked to determine ligand purity and as we presented in, Figure S1 TLR7^−/−^ iBMDMs are not responsive to R848, while LPS signaling via TLR4 was normal. Furthermore, WT and Mal^−/−^ iBMDMs showed similar expression of *Tlr7* and *Myd88* mRNA, indicating that observed *Ifnβ* gene expression is not impacted by perturbations in *Myd88* and *Tlr7* expression levels in the cell (Figure 1C).

Next, we attempted to analyze the role of Mal in the translational regulation of IFN-I. Thus, Mal^−/−^ and WT iBMDMs were stimulated with the TLR7 ligand R848 and TLR4 ligand-LPS, followed by IFN-I measurement in bioassay. Consistent with the hypothesis that Mal is a regulator of TLR7 mediated IFNβ induction, R848 treatment of Mal^−/−^ cells resulted in decreased production of IFN-I when compared to WT cells (Figure 1D). Correlating results of real-time PCR on LPS and R848 stimulation, IFN-I production was significantly decreased in MyD88^−/−^ iBMDMs when compared to WT (Figure 1D).

**Figure 2:**
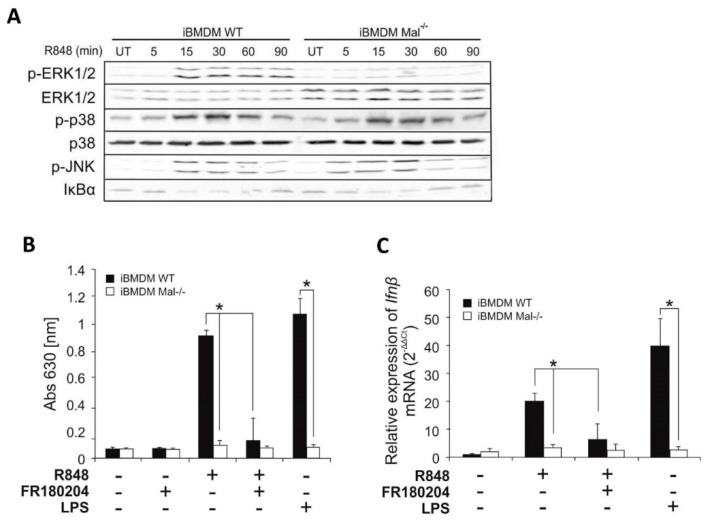
Mal regulates R848–induced ERK1/2 dependent activation of IFNβ. (**A**) Wild type and Mal^−/−^ iBMDMs were stimulated with R848 (100 nM) for indicated time periods. Cell lysates were subjected to SDS-PAGE. Protein detection was performed using specific antibodies and appropriate secondary antibodies conjugated to the fluorescent dye in the infrared range. Visualization was performed using the Odyssey CLx Imaging System LI-COR. The results presented are representative of at least three independent experiments. (**B**) WT and Mal^−/−^ iBMDMs were pretreated with DMSO (control) or FR180204 (2 µM) for 30 min. Next, cells were treated with R848 (100 nM) or LPS (100 ng/mL) for 16 h. Thereafter, type I IFN was measured by bioassay as described under “Materials and Methods.” Results are representative of at least three independent experiments performed in triplicate (Mean ± S.E.). (**C**) WT and Mal^−/−^ iBMDMs were pretreated with DMSO (control) or FR180204 (2 µM) for 30 min. Next, cells were treated with R848 (100 nM) or LPS (100 ng/mL) for 4 h. Thereafter, total RNA was isolated, converted to first-strand cDNA, and used as a template for quantitative real-time RT-PCR as described under “Materials and Methods.” Quantitative real-time PCR was used to assay the expression levels of *Ifnβ*. Experiments were repeated at least three times and data are presented in relative expression units where *Hprt* was used to normalize all samples and DMSO treated cells were assigned an arbitrary value of 1. * *p* ≤ 0.001.

In the next step, we assessed the ability of R848 to promote activation of classical signaling pathways in iBMDMs, as judged by phosphorylation or degradation of key regulatory proteins in Western blotting analysis. We observed only modest degradation of IκBα in response to R848, in contrast, TLR7 ligand promoted strong phosphorylation of the ERK1/2, p38, and JNK MAPK kinases, simultaneously impaired ERK1/2 phosphorylation in Mal^−/−^ iBMDMs could be observed (Figure 2A).

To explore the potential of ERK1/2 to mediate activation of IFNβ in response to R848, iBMDMs were pre-treated with the ERK1/2 inhibitor FR180204 [14] prior to stimulation with the TLR7 agonist and assessment of bioactive IFN-I and *Ifnβ* expression was performed by bioassay and quantitative PCR, respectively.

R848 promoted secretion of IFN-I by wild-type iBMDMs was strongly suppressed in cells pre-treated with FR180204 (Figure 2B) suggesting that ERK1/2 can positively regulate TLR7-induced IFN-I synthesis. Similarly, *Ifnβ* gene induction was evidently impaired in WT iBMDMs pre-treated with FR180204 following R848 stimulation (Figure 2C).

### 2.2. R848-Dependent Activation of IFNβ Requires IRF7 de Novo Synthesis

**Figure 3:**
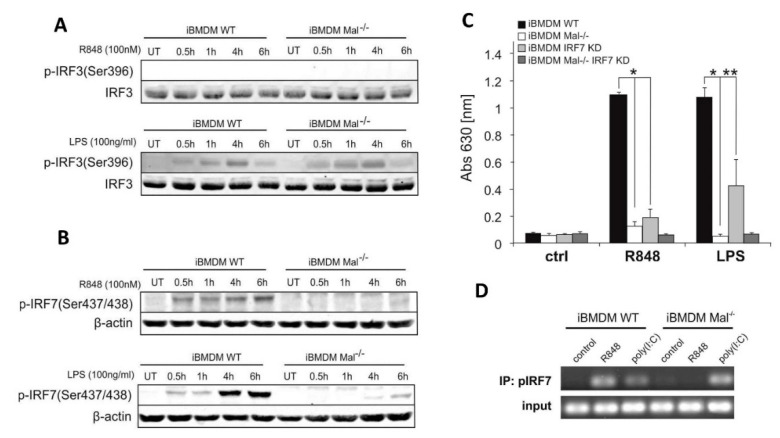
R848/TLR7/Mal-dependent activation of IFNβ is regulated by IRF7. (**A**,**B**) Wild type and Mal^−/−^ iBMDMs were stimulated with R848 (100 nM) or LPS (100 ng/mL) for the indicated time. Cell lysates were subjected to SDS-PAGE. Protein detection was performed using specific antibodies and appropriate secondary antibodies conjugated to the fluorescent dye in the infrared range. Visualization was performed using the Odyssey CLx Imaging System LI-COR. The results presented are representative of at least three independent experiments. (**C**) WT and Mal^−/−^ iBMDMs were transduced with shRNA specific for *Irf7* gene (IRF7 KD) or scrambled shRNA (control) (as described under “Materials and Methods”). Next, cells were treated with R848 (100 nM) or LPS (100 ng/mL) for 16 h. Type I IFN was measured by bioassay as described under “Materials and Methods.” Results are representative of at least three independent experiments performed in triplicate (Mean ± S.E.). (**D**) WT and Mal^−/−^ iBMDMs were treated with R848 (100 nM) or poly(I:C) (1 µg/mL) for 4 h. Cells were fixed in formaldehyde followed by nuclei isolation and sonication. Sonicated nuclear lysates were immunoprecipitated with an anti-IRF7 or rabbit IgG control antibody. Input DNA (prior to immunoprecipitation) and immunoprecipitated chromatin were analyzed by 35 cycles of standard PCR with primers designed to amplify an IFNβ coding sequence. * *p* ≤ 0.001; ** *p* ≤ 0.01.

To investigate the ability of Mal to modulate IFNβ induction at the transcriptional level WT and Mal^−/−^ iBMDMs were stimulated with the R848 (TLR7 ligand) and LPS (TLR4 ligand), followed by immunoblot analysis using an anti-phosphorylated IRF3 and IRF7 Ab to assess IRF3 and IRF7 phosphorylation status. As expected, LPS treatment induced the phosphorylation of both IRF3 and IRF7 in WT iBMDMs whereas in Mal^−/−^ iBMDMs only IRF3 phosphorylation was observed (Figure 3A,B lower panels). Notably, Mal-knockout evidently suppressed phosphorylation of IRF7 (Figure 3B, upper panel), whereas treatment with R848 resulted in phosphorylation of IRF7 in WT iBMDM only. As should be expected phosphorylation of IRF3 was not observed in both WT and Mal^−/−^ iBMDMs (Figure 3A upper panel). These findings suggest that R848 targets Mal-mediated activation of IRF7 but does not require IRF3. Moreover, supplementary results showed that this process is ERK kinase-dependent because after treatment of WT iBMDMs with ERK1/2 inhibitor, the phosphorylation of IRF7 is suppressed (Figure S2).

To investigate modulation IFNβ induction at the transcriptional level, we used the IFNβ, PDR I-III, Gal4-IRF7, and Gal4-IRF3 luciferase reporter constructs. HEK293/TLR7 cells were transfected with an increasing amount of the expression vector encoding Mal protein. Interestingly, we found that transfection of HEK293/TLR7 with the expression plasmid encoding Mal upregulated R848-induced activation of IFNβ and PRD I-III reporter genes (Figure S3A,B). We next addressed the question if the related transcriptional regulator IRF was a target of TLR7 signaling. IRF7 and IRF3 activation was measured in dual-luciferase assay using a Gal4–IRF7 or Gal4–IRF3 fusion protein. As we have shown, only Mal cotrasfection of HEK293/TLR7 cells together with R848 treatment led to abundant IRF7 coactivation (Figure S3C,D) and indicated that Mal/IRF7 plays role in the regulation of IFNβ expression.

Next, to confirm the role of IRF7 in the regulation of TLR7-induced production of IFN-I we then infected iBMDMs with lentiviral particles containing control or IRF7-specific shRNA.). Knockdown of *Irf7* resulted in strong inhibition of R848-induced secretion of IFN-I, indicating a specific regulatory role of IRF7 in R848-induced production of IFN-I (Figure 3C).

**Figure 4:**
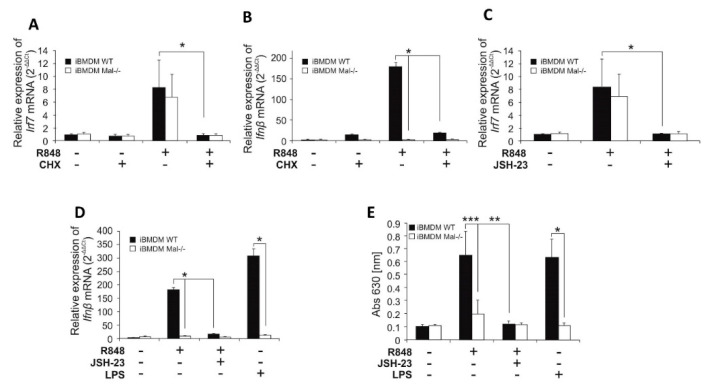
R848/Mal-dependent production of IFNβ requires de novo IRF7 biosynthesis via NF-κB-dependent, but Mal-independent, mechanism. (**A**,**B**) Wild type and Mal^−/−^ iBMDMs were pretreated with DMSO (control) or cycloheximide, CHX (10 µM) for 30 min. Next, cells were treated with R848 (100 nM) for 4 h. Thereafter, total RNA was isolated, converted to first-strand cDNA, and used as a template for quantitative real-time RT-PCR as described under “Materials and Methods.” Quantitative real-time PCR was used to assay the expression levels of *Irf7* (**A**) and *Ifnβ* (**B**). Experiments were repeated at least three times and data are presented in relative expression units where *Hprt* was used to normalize all samples and DMSO treated cells were assigned an arbitrary value of 1. (**C**–**E**) WT and Mal^−/−^ iBMDMs were pretreated with DMSO (control) or JSH-23 (10 µM) for 30 min. Next, cells were treated with R848 (100 nM) or LPS (100 ng/mL) for 4 h (**C**,**D**) or 16 h (**E**). (**C**,**D**) Total RNA was isolated, converted to first-strand cDNA, and used as a template for quantitative real-time RT-PCR as described under “Materials and Methods.” Quantitative real-time RT-PCR was used to assay the expression levels of *Irf7* (**C**), *Ifnβ* (**D**). Experiments were repeated at least three times and data are presented in relative expression units where *Hprt* was used to normalize all samples and DMSO treated cells were assigned an arbitrary value of 1. (**E**) Type I IFN was measured by bioassay as described under “Materials and Methods.” Results are representative of at least three independent experiments performed in triplicate (Mean ± S.E.). * *p* ≤ 0.001; ** *p* ≤ 0.01; *** *p* ≤ 0.05.

Given such clear evidence of IRF7 role in the regulation of *Ifnβ* expression, we treated iBMDMs from WT and Mal^−/−^ mice with R848 or poly(I:C) and then ex vivo binding of IRF7 to the IFNβ promoter was assayed by chromatin immunoprecipitation. R848 promoted strong binding of IRF7 to the IFNβ promoter in WT but not in Mal^−/−^ iBMDMs (Figure 3D). In contrast, as previously shown, when stimulating with poly(I:C) modest binding of IRF7 to the IFNβ promoter in WT cells was observed, but interaction was considerably enhanced in Mal^−/−^ cells. We then addressed the question of whether the effect of IRF7 on TLR7-induced expression of IFNβ is mediated by direct recruitment of IRF7 to the IFNβ promoter or indirectly by induction of de novo expression of IRF7. We treated WT and Mal^−/−^ iBMDMs with the protein synthesis inhibitor–cycloheximide and studied its effect on R848-induced expression of *Ifnβ* and *Irf7* mRNA (Figure 4A,B). Cycloheximide reduced the ability of R848 to induce mRNA expression for both genes, which confirmed that iBMDMs after TLR7 activation required IRF7 biosynthesis to ensure IFNβ promoter activation. Furthermore, no difference was observed in IRF7 mRNA expression between WT and Mal^−/−^ iBMDMs (Figure 4A), suggesting that activation of *Irf7* gene transcription by R848 is Mal-independent.

### 2.3. NF-κB Positively Regulates R848-Induced Expression of Irf7 mRNA

Next, we sought to investigate the role of NF-κB in the transcriptional regulation of *Irf7* mRNA and IRF7-dependent gene-*Ifnβ*. To explore the potential of NF-κB to initiate *Ifnβ* gene expression after the activation of TLR7, WT and Mal^−/−^ iBMDMs previously pretreated with the NF-κB inhibitor-JSH-23 [15], were stimulated with the TLR7 agonist R848, and the expression of *Irf7* mRNA was assayed by quantitative PCR (Figure 4C). R848 promoted similar induction of *Irf7* expression in both WT and Mal^−/−^ iBMDMs, that was strongly suppressed in cells pretreated with JSH-23, suggesting that NF-κB can positively regulate R848-induced *Irf7* gene expression through a Mal-independent mechanism. The negative effect of JSH-23 on *Irf7* gene expression specifically applies to *Ifnβ* expression (Figure 4D) and IFN-I secretion (Figure 4E) as JSH-23 was able to inhibit R848-induced IFNβ expression and secretion.

### 2.4. TLR7 Induced IP-10 Expression Is Regulated by Mal/Erk-Dependent Pathway

**Figure 5:**
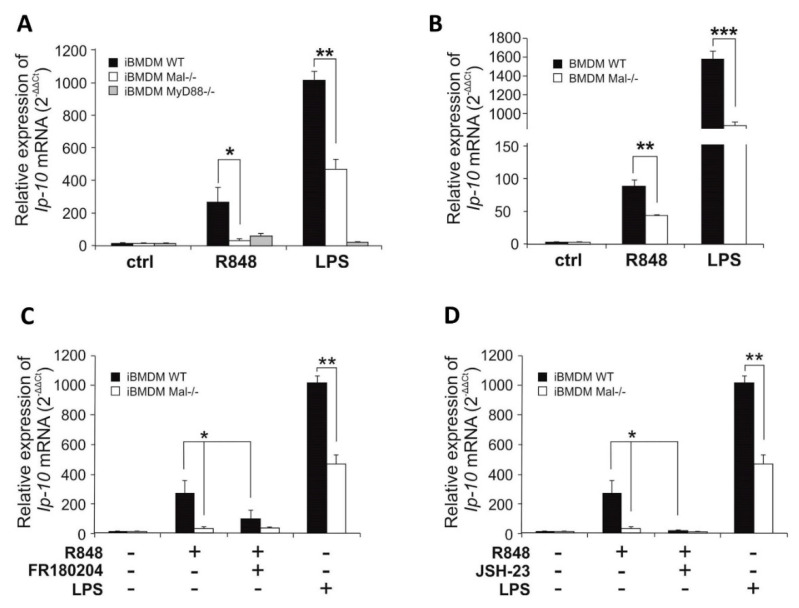
Mal/ERK1/2–dependent *IFNβ* expression results in *Ip-10* upregulation. (**A**,**B**) WT, Mal ^−/−^ and MyD88^−/−^ iBMDMs (**A**) and macrophages isolated from bone marrow of wild type mice (BMDM WT) and Mal deficient mice (BMDM Mal^−/−^) (**B**) were treated with R848 (100 nM) or LPS (100 ng/mL) for 4 h. Thereafter, total RNA was isolated, converted to first-strand cDNA, and used as a template for quantitative real-time RT-PCR as described under “Materials and Methods.” Quantitative real-time RT-PCR was used to assay the expression levels of *Ip-10*. Experiments were repeated at least three times and data are presented in relative expression units where *Hprt* was used to normalize all samples and non-treated cells were assigned an arbitrary value of 1. (**C**,**D**) WT and Mal^−/−^ iBMDMs were pretreated with DMSO (control), FR180204 (2 µM) (**C**), or JSH-23 (10 µM) (**D**) for 30 min. Next, cells were treated with R848 (100 nM) or LPS (100 ng/mL) for 4 h. Thereafter, total RNA was isolated, converted to first-strand cDNA, and used as a template for quantitative real-time RT-PCR as described under “Materials and Methods.” Quantitative real-time RT-PCR was used to assay the expression levels of *Ip-10*. Experiments were repeated at least three times and data are presented in relative expression units where *Hprt* was used to normalize all samples and non-treated cells were assigned an arbitrary value of 1. * *p* ≤ 0.001; ** *p* ≤ 0.01; *** *p* ≤ 0.05.

Considering that IFN-I drives the expression of IP-10 [16], in the next step we examined the ability of Mal to modulate IFN dependent gene *Ip-10*. We initially assessed the ability of Mal adapter to modulate TLR7-mediated IP-10 production by measurement of R848-induced *Ip-10* mRNA expression by quantitative real-time PCR. Results demonstrated that whereas stimulation of WT iBMDMs with the TLR7 ligand resulted in *Ip-10* gene induction, a significantly lower induction of *Ip-10* was evident in Mal^−/−^ iBMDMs. Impaired *Ip-10* gene induction was also observed in Mal^−/−^ iBMDMs compared with WT controls following LPS stimulation (Figure 5A). A similar effect of Mal on *Ip-10* gene expression was observed in macrophages in vitro differentiated from WT and Mal^−/−^ mice bone marrow (WT and Mal^−/−^ BMDMs) (Figure 5B).

To explore the potential of ERK1/2 and NF-κB to mediate expression of *Ip-10* in response to R848 sensing, iBMDMs were pre-treated with the ERK1/2 inhibitor-FR180204 or NF-κB inhibitor-JSH-23 prior to stimulation with the TLR7 agonist and analysis of *Ip-10* expression by quantitative PCR was performed (Figure 5C,D).

R848 promoted expression of *Ip-10* in WT iBMDMs and this effect was strongly suppressed in cells pre-treated with FR180204 (Figure 5C) or JSH-23 (Figure 5D) suggesting that ERK1/2 and NF-κB positively regulate TLR7-induced *Ip-10* expression.

### 2.5. Blocking of Mal in Human Dendritic Cell Line Decreases R837-Induced Expression of IFNβ and IP-10

**Figure 6:**
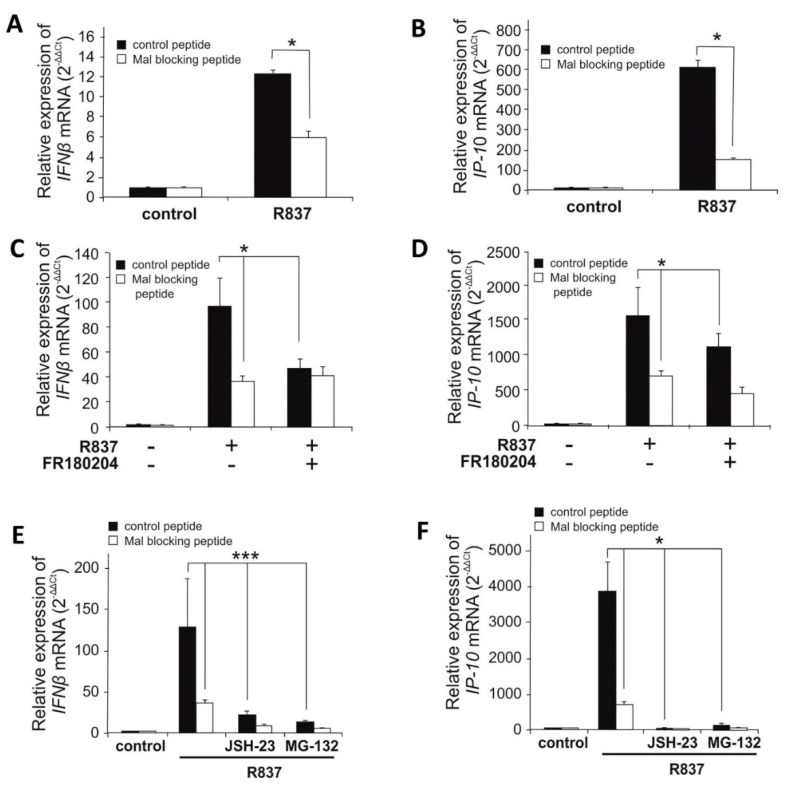
Selective agonist of TLR7 induces expression of IFNβ and *IP-10* in human pmDC05 cell line in Mal dependent manner. Human pmDC05 cells were pretreated with control or Mal blocking peptide (20 µM) for 16h. Next, cells were pretreated with DMSO (control), FR180204 (2 µM) (**C**,**D**) or JSH-23 (10 µM) and MG-132 (5 µM) (**E**,**F**) for 30 min. Thereafter, cells were treated with R837 (10 µg/mL) for 4 h. Total RNA was isolated, converted to first-strand cDNA, and used as a template for quantitative real-time RT-PCR as described under “Materials and Methods.” Quantitative real-time RT-PCR was used to assay the expression levels of *IFNβ* (**A**,**C**,**E**), *IP-10* (**B**,**D**,**F**). Experiments were repeated at least three times and data are presented in relative expression units where *HPRT* was used to normalize all samples and DMSO treated cells (control) were assigned an arbitrary value of 1. * *p* ≤ 0.001; *** *p* ≤ 0.05.

To negate the possibility of species-dependent differences in Mal functionality in the context of TLR7 signaling, the ability of Mal to regulate TLR7 signaling in a human system was investigated. Thus, the human dendritic cell line was chosen as model cells considering their pronounced TLR7 expression and robust responsiveness to a selective TLR7 agonist - R837. Following the suppression of Mal by specific blocking peptide, we assessed *IFNβ* and *IP-10* induction after R837 stimulation. It was shown that blocking of Mal significantly decreased R837-induced *IFNβ* and *IP-10* gene induction in human dendritic cells (Figure 6A,B) in comparison to cells treated with control peptide. Next, we sought to investigate the role of ERK1/2 and NF-κB in the modulation of TLR7-dependent induction of *IFNβ* and *IP-10*. Dendritic cells were pretreated with ERK1/2 inhibitor-FR180204 (Figure 6C,D) and two NF-κB inhibitors: JSH-23 or MG-132 (Figure 6E,F) in the absence or presence of Mal inhibitory peptide, followed by measurement of *IFNβ* and *IP-10* gene induction. It was found that suppression of both ERK1/2 and NF-κB pathways significantly decreased TLR7-induced *IFNβ* and *IP-10* expression. According to results obtained from iBMDM (Figure 2B,C) it could be concluded that attenuation of R848-induced *IFNβ* expression by ERK1/2 inhibitor is Mal dependent (Figure 6C,D). As shown in Figure 2A, NF-κB activation by R848 is a Mal-independent process. However, this pathway is needed for de novo synthesis of IRF7, that in turn is required for Mal-dependent expression of *IFNβ*. Therefore, despite the Mal is dispensable in this mechanism, a strong decrease in R848-induced *IFNβ* and *IP-10* expression was observed in response to inhibition of the NF-κB pathway (Figure 6E,F).

## 3. Discussion

Toll–like receptors are pivotal members of the host’s innate immune response and instigate a response against both bacteria and viruses. Activation of TLR signaling by synthetic ligands leads to the expression of genes encoding type I IFNs, including IFNβ and chemokines i.e., IP-10. Expression of these effector proteins depends on the activation of a number of transcription factors such as NF-κB and IRFs. This process is mediated by various adaptor proteins including MyD88, Mal/TIRAP, TRIF, and TRAM. Although much light has been shed on the mechanisms behind TLR-dependent signaling, published data still question the role of the adaptor protein Mal in signaling pathways activated by intracellular Toll–like receptors [10,11,17,18,19].

Here, we investigate the role of the adaptor protein Mal in TLR7-dependent signaling. We show for the first-time molecular mechanism, that TLR7-activated IFNβ expression is impaired in Mal–deficient murine macrophages and human plasmacytoid dendritic cells treated with the Mal inhibitory peptide. Convergent observation was published by Bonham et al. [10], authors investigated levels of IFNα secreted by murine macrophages exposed to the influenza virus and concluded a Mal-dependent expression profile [10]. However, a whole viral particle can be recognized not only by different Toll–like receptors [20] but can engage a set of intracellular PRRs such as PKR or RIG-I [21], thus our observation that Mal/Erk/IRF7 path is required for R848-dependent signaling is a novelty.

We also demonstrate that TLR7–induced IFNβ expression requires ERK1/2 activation and that this process is also abolished in Mal^−/−^ cells. In accordance with Honda et al. [22], we report that IRF7 is a key transcription factor regulating the expression of IFNβ. Notably, we show for the first time that the R848-induced IRF7 activation is abolished in Mal^−/−^ iBMDMs, importantly this finding correlates with the IRF7 inability to bind to the IFNβ promoter region in Mal-deficient macrophages upon R848 treatment. Surprisingly, we also found that although IRF7 is expressed constitutively at a very low level in macrophages [23], it is synthesized de novo following TLR7 activation in a Mal-independent manner and that this phenomenon has a direct impact on R848-induced cellular response, as the inhibition of protein translation abrogates TLR7-dependent IFNβ expression. It is known, that *Irf7* gene expression can be elevated by factors such as cytokines, type I IFN, or LPS [24]. Moreover, we show that inhibition of NF-κB transcription factor abolishes R848-induced expression of IRF7. It is rather plausible that the R848-induced increase in IRF7 levels is a direct effect of TLR7 engagement, as it has been shown that the *Irf7* promoter region contains at least four NF-κB binding sites [23]. It was also shown that the IRF7 half-life in murine splenocytes and thymocytes reaches approximately four hours [25]. Moreover, the IRF7 transcription factor’s aa sequence is rich in Pro-Glu-Ser-Thr repeats which targets protein for proteasomal degradation. In conclusion, these data suggest that the inhibition of NF-κB activity results in the abolished IRF7 expression, which diminishes the TLR7-dependent IFNβ expression.

We also report that Mal is indirectly involved in regulating the TLR7-dependent *Ip-10* expression and the loss of *Ip-10* expression in Mal-deficient cells can be attributed to the impaired R848-induced IFNβ expression.

The presented study fails to indicate the precise mechanism in which the adaptor protein Mal regulates TLR7-induced IFNβ expression in both murine macrophages and human plasmacytoid dendritic cells. However, CoIP experiments on HEK293 transfected with Mal-HA and TLR7-Flag indicate the interaction of these proteins (Figure S4). It is plausible that the TIR domain of the adaptor protein interacts directly with the homological domain of the TLR7 receptor. It has been previously shown that Mal coprecipitates with the MyD88 and IRAK4 proteins following TLR9 activation [19]. Due to the homology level between TLR7 and TLR9, it seems that Mal should play a strictly structural role in TLR7–dependent signaling. This notion is also supported by the results from experiments with the Mal blocking peptide, taking into account that it interacts directly with the receptor’s TIR domain blocking further signal transduction. Moreover, Mal adaptor protein does not have any catalytic properties, thus we speculate that it may stabilize the MyD88/TRAF6/IRAK1/IRAK4/IRF7 complex formed after TLR7 activation in a way similar to the iOPN (intracellular osteopontin) protein. iOPN has been shown to be crucial for IRF7 activation in plasmacytoid dendritic cells [26] and Mal has been shown to interact directly with IRF7 but not IRF3 [9].

Recent studies regarding whole-genome sequencing of SARS-CoV, MERS-CoV, and SARS-CoV-2 has demonstrated that the SARS-CoV-2 genome contains many ssRNA motifs that could interact with TLR7, indicating that TLR7 signaling might be relevant in the pathogenesis of COVID-19 [27] and our study provides an insight into the molecular mechanisms of the anti-viral innate immune response activated by TLR7.

In conclusion, our results indicate that following R848 stimulation the signaling pathways lead to the Mal/ERK/IRF7 dependent activation of IFNβ expression and the Mal–independent path involving NF-κB activation of IRF7 expression. By identifying Mal as a critical positive regulator of the R848-dependent IFNβ induction.

## 4. Materials and Methods

### 4.1. Cell Culture and Reagents

Immortalized BMDM cell lines from wild type (iBMDM WT), Mal^−/−^ (iBMDM Mal^−/−^), MyD88^−/−^ (iBMDM MyD88^−/−^) and TLR7^−/−^ (iBMDM TLR7^−/−^) mice were obtained from Bei Resources (Manassas, VA, USA). BMDM were differentiated in vitro from bone marrow cells. In detail, bone marrow was isolated from femurs and tibiae of Mal-deficient mice and their wild-type littermates and cultured in medium (DMEM high glucose supplemented with 10% (*v*/*v*) FCS, 2 mM L-glutamine and Normocin) in T75 flasks. For differentiation cells into BMDMs in culture, medium was supplemented with 20% (*v*/*v*) of supernatant taken from MCSF-L929 cells (a murine M-CSF-producing cell line) for 7 days. pmDC05 cell line was generated by Miwako Narita, Niigata University. Cells were grown in IMDM with L-Glutamine and 25 mM HEPES (Gibco, Gaithersburg, MD, USA) (pmDC05) and in DMEM with GlutaMAX (Gibco, Gaithersburg, MD, USA) (iBMDMs and BMDMs) supplemented with 10% heat-inactivated fetal bovine serum (Sigma, St. Louis, MO, USA) and Normocin (Invivogen, San Diego, CA, USA) and maintained in a humidified atmosphere of 5% CO2. Ultra-pure LPS-EB derived from *E. coli* strain O111:B4, poly (I:C), R848, and R837 were purchased from Invivogen (San Diego, CA, USA). Inhibitors FR180204, JSH-23, MG-132, and cycloheximide were purchased from Sigma (St. Louis, MO, USA). Control and Mal inhibitory peptides were from Novus (Manchester, UK). All animal protocols used in this study were approved by the Ethical Committee at the Institute of Immunology and Experimental Therapy, Polish Academy of Sciences, Wroclaw.

### 4.2. Type I IFN Bioassay

Mouse cells were seeded (5 × 10^5^ cells/mL; 200 µL) in 96-well plates and stimulated as indicated. Detection of bioactive murine type I IFN was assessed using B16-Blue IFN-α/β cells, essentially as described by the manufacturer (Invivogen, San Diego, CA, USA).

### 4.3. Chromatin Immunoprecipitation Assay

WT and Mal^−/−^ iBMDMs were grown to confluency in 6-well plates and stimulated with 100 nM R848 or 1 µg/mL poly(I:C) for 4h. The immunoprecipitation procedure was performed as previously described [15] with anti-phospho-IRF7 antibody (Biorbyt, Cambridge, UK). Standard PCR was conducted with specific primers designed to amplify a region of the IFNβ coding sequence. The primers were as follows: forward: 5′-GGAGATGACGGAGAAGATGC-3′ and reverse: 5′-CCCAGTGCTGGAGAAATTGT-3′. PCR products were resolved by 1.5% (*w*/*v*) agarose gel electrophoresis and then analyzed using a Gel Doc (BioRad, Hercules, CA, USA).

### 4.4. Lentiviral Transduction

Scrambled shRNA and murine IRF7 lentiviral shRNA plasmids were from Sigma and the procedure was followed as described [9]. WT and Mal^−/−^ iBMDMs (1 × 10^5^ cells/well; 6 well plates) were transduced with scrambled shRNA or mIRF7 shRNA lentiviral particles and cells subsequently grown for 1 week under puromycin (10 μg/mL) selection. The efficiency of IRF7 knockdown was assessed by RT-PCR using the following primers: *Irf7*, forward: CCCATCTTCGACTTCAGC and reverse: GACACACCCTCACGCTGC; *Hprt*, forward: GCTTGCTGGTGAAAAGGACCTCTCTCGAAG and reverse: CCCTGAAGTACTCATTATAGTCAAGGGCAT.

### 4.5. First-Strand cDNA Synthesis

BMDMs, iBMDMs, and pmDC05 were seeded (1 × 10^6^ cells/mL, 1mL) in 6-well plates and grown for 24 h. Cells were then pretreated for 30 min with 2 µM FR180204, 5 µM MG-132, 10 µM cycloheximide or 10 µM JSH-23 prior to stimulation for 4 h with 100 nM R848, 10 µg/mL R837 or 100 ng/mL LPS-EB. Total RNA was isolated using ReliaPrep (Promega, Madison, WI, USA) according to the manufacturer’s protocol. Isolated RNA (1µg) was incubated with random hexamer primers (1 µL; 500 µg/mL) at 70 °C for 5 min. Thereafter, the other reaction components were added in the following order: 5 µL of 5xRT buffer, 1.3 µL of 10 mM dNTP, 1µL of MMLV Reverse transcriptase (Promega, Madison, WI, USA), and nuclease-free water to a total volume of 25 µL. Reactions were incubated at 37 °C for 40 min followed by 42 °C for 40 min and heating to 80 °C for 5 min.

### 4.6. PCR and qPCR

Total cDNA (10 ng for iBMDMs and BMDMs and 5 ng for pmDC05) was used as starting material for qPCR with CFX Connect qPCR system (BioRad, Hercules, CA, USA) and GoTaq qPCR Master mix (Promega, Madison, WI, USA) with dNTPs, 0.5 µM each. For the amplification of the specific genes the following primers were used: *Ifnβ*, forward: GGAGATGACGGAGAAGATGC and reverse: CCCAGTGCTGGAGAAATTGT; *IFNβ*, forward: GCCGCATTGACCATCTATGA and reverse: GCCAGGAGGTTCTCAACAATAG; *Ip-10*: forward, GCCATGGTCCTGAGACAAA and reverse: AGCTTACAGTACAGAGCTAGGA; *IP-10*, forward: GGAGATGAGCTAGGATAGAGGG and reverse: TGCCCATTTTCCCAGGACCG. For each mRNA quantification, the housekeeping gene hypoxanthine phosphoribosyltransferase 1 (HPRT) was used as a reference point using the following primers: *Hprt*, forward: GCTTGCTGGTGAAAAGGACCTCTCTCGAAG and reverse: CCCTGAAGTACTCATTATAGTCAAGGGCAT; *HPRT*, forward: AGCTTGCTGGTGAAAAGGAC and reverse: TTATAGTCAAGGGCATATCC. Real-time PCR data were analyzed using 2^−ΔΔCT^ method. Conventional PCR was performed using DNA RedTaq polymerase (Sigma) with 70 ng of total cDNA according to the manufacturer’s protocol. For the amplification of the specific genes the following primers were used: *Mal*, forward: AGCGGAGAACAATCGCTCTACCAA and reverse: AGATCGGCATCTTCTTGGGCTTCT; *Myd88*, forward: TTCAGCATTTGGGAGGTAGAGGCA and reverse: GCGAAGCCAAACAGCTTCTCCTTT; *Tlr7*, forward: GCCATCCAGCTTACATCTTCT and reverse: TTTGACCCAGGTAGAGTGTTTC; *Trif*, forward: GGACCTCAGCCTCTCATTATTC and reverse: CTCCGAACACTCAGTCTTG; *Tram*, forward: TCTCAATCACCGAATGGTAAGG and reverse: GCAGACGAGGGAGCTTTATT; *Hprt* (sequence above) used as housekeeping gene of reference. PCR products were resolved by 1.5% (*w*/*v*) agarose gel electrophoresis and then analyzed using a Gel Doc (BioRad, Hercules, CA, USA).

### 4.7. Western Blotting

WT and Mal^−/−^ iBMDMs were seeded (1 × 10^6^ cells/mL; 2mL) on a 6-well plate and grown for 24 h. Cells were then treated with 100 nM R848 or 100 ng/mL LPS for designated times. Cells were washed in ice-cold PBS and lysed in HS buffer. Cell lysates were subjected to SDS-PAGE followed by Western blot analysis with an anti-phospho-ERK1/2, anti-ERK1/2, anti-phospho-p38, anti-p38, anti-phospho-JNK, anti-phospho-IRF3, anti-IRF3, anti-phospho-IRF7 (Cell Signaling, Danvers, MA, USA), anti-β-actin (Sigma, St. Louis, MO, USA), and anti-IκBα (Santa Cruz Biotechnology, Dallas, TX, USA) antibody, secondary antibodies: IRDye 800CW Goat anti-Rabbit IgG (H + L), IRDye 680RD Donkey anti-Mouse IgG (H + L) (LI-COR, Lincoln, NE, USA). Imaging was performed using ODYSSEY CLx Infrared Imaging System (LI-COR).

### 4.8. Data Analysis

Statistical analysis was carried out using the unpaired Student’s *t*-test using SigmaPlot 2001 program (Systat Software, San Jose, CA, USA). *P*-values of less than or equal to 0.05 were considered to indicate a statistically significant difference where * indicated *p* ≤ 0.001; ** *p* ≤ 0.01; *** *p* ≤ 0.05.

## Supplementary Materials

The following are available online at https://www.mdpi.com/1422-0067/21/23/8925/s1.

## Abbreviations

BMDM	Macrophages in vitro differentiated from mice bone marrow
iBMDM	Immortalized bone marrow-derived macrophages from primary murine bone marrow cells
IFNβ	Interferon-beta
IP-10	Interferon gamma-induced protein 10
IRF7	Interferon regulatory factor 7
Mal/TIRAP	MyD88 adaptor-like/TIR adaptor protein
R837	Imiquimod
R848	Resiquimod
TLR7	Toll-like receptor 7
