# Supplementary material for: Absence of Mal/TIRAP Results in Abrogated Imidazoquinolinones-Dependent Activation of IRF7 and Suppressed IFNβ and IFN-I Activated Gene Production
Source: Int J Mol Sci. 2020 Nov 25;21(23):8925. doi: 10.3390/ijms21238925 (PMC7727842; doi:10.3390/ijms21238925)
Supplement: Supplementary file 1 [file ijms-21-08925-s001.pdf]

International Journal of
Molecular Sciences

MDPI

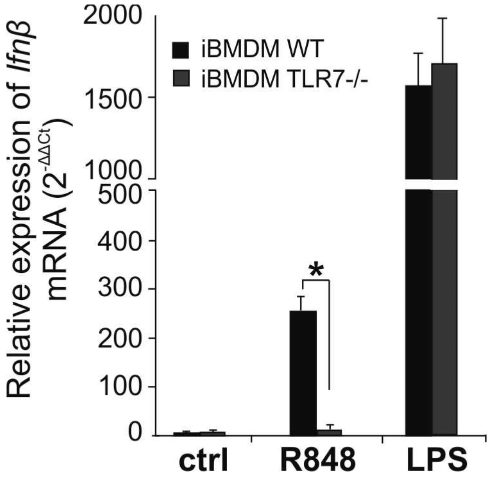

Figure S1. Expression of Ifnβ in LPS-induced WT and TLR7$^{-/-}$ iBMDMs. WT and TLR7$^{-/-}$ iBMDMs were treated with R848 (1μM) or LPS (1μg/ml) for 4 hr. Thereafter, total RNA was isolated, converted to first-strand cDNA and used as a template for quantitative real-time PCR. Real-time PCR was performed using GoTaq qPCR Master mix (Promega) with 5 ng of total cDNA according to the manufacturer's protocol. For the amplification of the Ifnβ gene the following primers were used: forward: GGAGATGACGGAGAAGATGC and reverse: CCCAGTGCTGGAGAAATTGT. For mRNA quantification, the housekeeping gene hypoxanthine phosphoribosyltransferase 1 (HPRT) was used as a reference point using the following primers: Hprt, forward: GCTTGCTGGTGAAAAGGACCTCTCTCGAAG and reverse: CCCTGAAGTACTCATTATAGTCAAGGGCAT. Real-time PCR data were analyzed using $2^{-\Delta\Delta CT}$ method. * $p<0.001$.

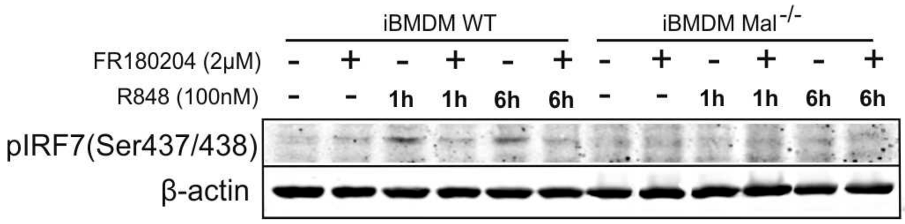

Figure S2. ERK1/2 mediate Mal dependent phosphorylation of IRF7 in response to R848. Wild type and Mal$^{-/-}$ iBMDMs were pretreated with FR180204 (2 μM) for 30 min. Next, cells were stimulated with R848 (100 nM) for the indicated time. Cell lysates were subjected to SDS-PAGE. Protein detection was performed using specific antibodies: anti-phospho-IRF7 (Cell Signaling) and anti-β-actin (Sigma), and appropriate secondary antibodies conjugated to the fluorescent dye in the infrared range: IRDye 800CW Goat anti-Rabbit IgG (H + L), IRDye 680RD Donkey anti-Mouse IgG (H + L) (LI-COR). Visualization was performed using the Odyssey CLx Imaging System LI-COR.

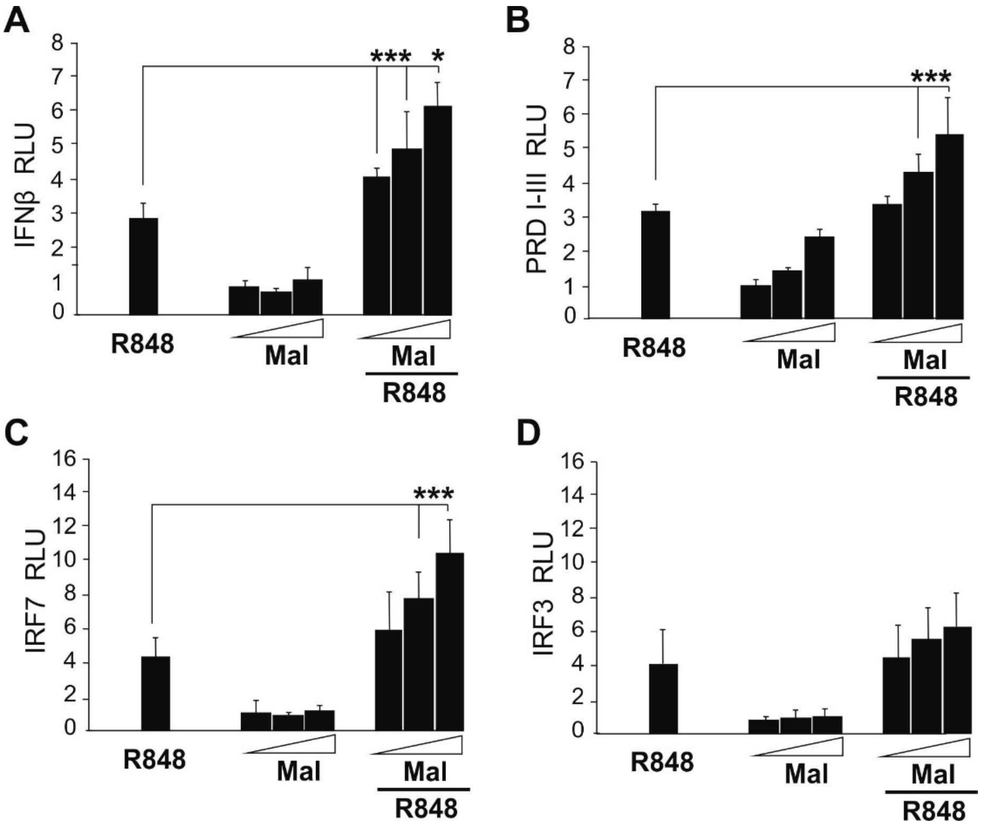

Figure S3. TLR7 dependent activation of Ifnβ gene by R848 is regulated by Mal/ phosphorylation of IRF7/ activation of PRD I-III domain of IFNβ promoter. HEK293/TLR7 cells were co-transfected with vectors encoding a reporter gene for the full length IFNβ promoter (A), IFNβ PRD I-III (B), IRF7-Gal4 (C) or IRF3-Gal4 (D) and co-transfected with increasing amounts of the expression vector encoding Mal (1, 10, 20 ng) as indicated. After 24 hr cells were stimulated with R848 (100 nM) as indicated. After 16 hr lysates were harvested followed by assessment of dual-luciferase gene reporter activity. *p<0.001; ***p<0.05.

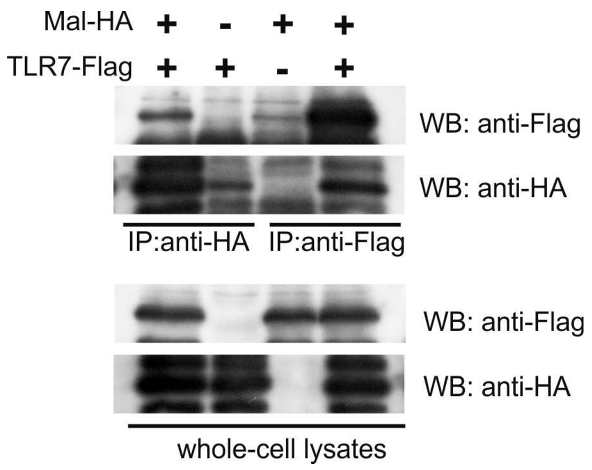

Figure S4. Co-immunoprecipitation of HA-Mal with Flag-IRF7. HEK293 cells were co-transfected with plasmid encoding Mal-HA and TLR7-Flag or EmptyVector as indicated. After 24 hr, immunoprecipitation (IP) of the HA-tag or Flag-tag was performed using an antibody as indicated. Cell lysates were subjected to SDS-PAGE. Immunoblot analysis was performed using an anti-Flag M1 antibody (Sigma) and anti-HA 101r antibody (Covance). Immunoblot analysis of whole cell lysates was performed to confirm the equal expression of the indicated proteins within each sample. Visualization was performed using a Gel Doc (BioRad).
